# Zn^2+^ reverses functional deficits in a *de novo* dopamine transporter variant associated with autism spectrum disorder

**DOI:** 10.1186/s13229-015-0002-7

**Published:** 2015-02-24

**Authors:** Peter J Hamilton, Aparna Shekar, Andrea N Belovich, Nicole Bibus Christianson, Nicholas G Campbell, James S Sutcliffe, Aurelio Galli, Heinrich JG Matthies, Kevin Erreger

**Affiliations:** Department of Molecular Physiology and Biophysics, Vanderbilt University School of Medicine, Room 7124 MRB III, 465 21st Avenue S, Nashville, TN 37232 USA; Vanderbilt Brain Institute, Vanderbilt University School of Medicine, Nashville, Tennessee USA; Department of Pharmacology, Vanderbilt University School of Medicine, Nashville, Tennessee USA

**Keywords:** Autism spectrum disorder, Dopamine, Zinc, Transporter, *de novo* mutation

## Abstract

Our laboratory recently characterized a novel autism spectrum disorder (ASD)-associated *de novo* missense mutation in the human dopamine transporter (hDAT) gene SLC6A3 (hDAT T356M). This hDAT variant exhibits dysfunctional forward and reverse transport properties that may contribute to DA dysfunction in ASD. Here, we report that Zn^2+^ reverses, at least in part, the functional deficits of ASD-associated hDAT variant T356M. These data suggest that the molecular mechanism targeted by Zn^2+^ to restore partial function in hDAT T356M may be a novel therapeutic target to rescue functional deficits in hDAT variants associated with ASD.

## Findings

### Introduction

The dopamine (DA) transporter (DAT) tunes DA neurotransmission by active re-uptake of DA from the synapse [1]. Our laboratory has recently characterized the first *de novo* mutation in the human dopamine transporter (hDAT) reported in a patient diagnosed with autism spectrum disorder (ASD), which results in a Thr to Met substitution at site 356 (hDAT T356M). We reported novel and profound functional abnormalities associated with the hDAT *de novo* mutation T356M, resulting in enhancement of non-vesicular, DAT-dependent DA release, referred to as anomalous DA efflux. Our data raise the possibility that anomalous DA efflux (or other disturbances in DA neurotransmission) may represent a complication relevant for behavioral abnormalities in ASD. Extracellular zinc (Zn^2+^) inhibits DA uptake [2]. Three amino acid side chains have been identified in DAT which coordinate zinc: H193 in extracellular loop 2 (EL2), H375 in the first helical part of extracellular loop 4 (EL4A), and E396 in the second helix of extracellular loop 4 (EL4B) [2,3]. Structural data from the DAT-homolog LeuT in the inward- and outward-facing conformation suggest that the relative orientation of H375 and E396 shifts during the transport cycle [4].

hDAT T356M displays decreased forward and reverse-transport function compared with wild-type hDAT [5]. The reduced transport capacity of the mutant was not associated with a reduction in hDAT surface expression. Amphetamine (AMPH) is a psychostimulant that targets the hDAT causing reverse transport of DA (DA efflux) [6]. hDAT T356M exhibits impaired AMPH-induced DA efflux. Here, we show that the presence of Zn^2+^ partially rescues both DA uptake and the AMPH-induced DA efflux impairments of hDAT T356M. Zn^2+^ diminishes the anomalous DA efflux property of the hDAT T356M, which might account for its ability to partially rescue transporter functions. Rescue of hDAT function by Zn^2+^ might reveal a new molecular mechanism to target for pharmacological intervention in patients with ASD.

## Results

### Zn^2+^ enhances [^3^H]DA uptake in hDAT T356M

**Figure 1:**
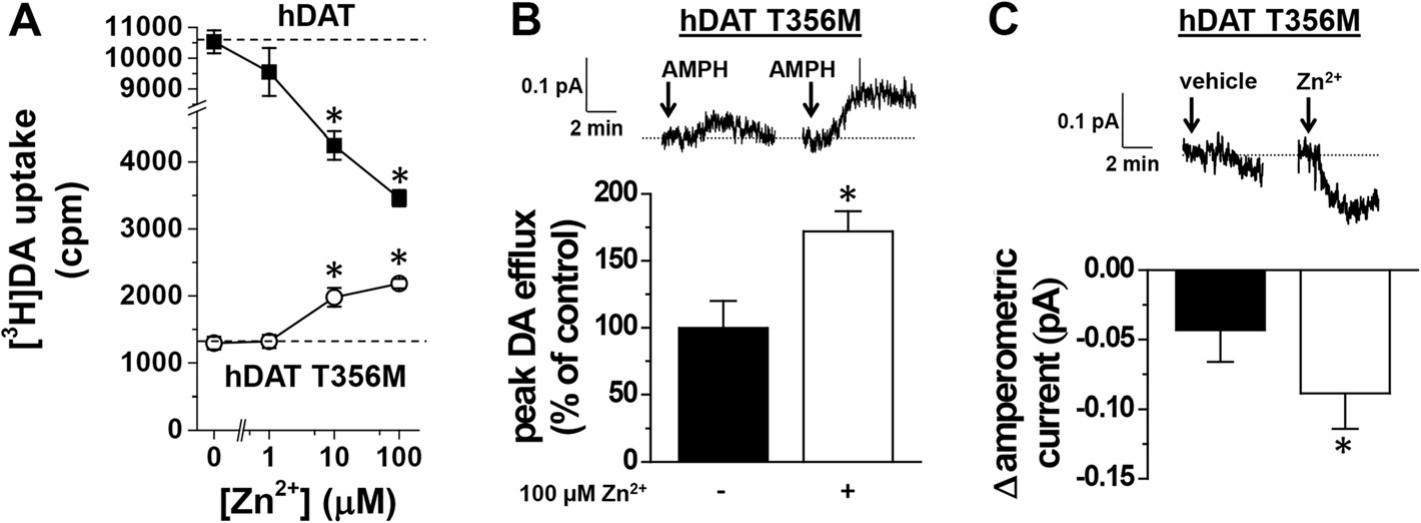
**Zn** ^**2+**^ **partially reverses the hDAT T356M deficits in [** ^**3**^ **H]DA uptake and amphetamine (AMPH)-mediated efflux.** Methods were as previously described in Hamilton et al. [5]. **(A)** [^3^H]DA uptake counts (cpm) are plotted for hDAT and hDAT T356M over a range of Zn^2+^ concentrations. While Zn^2+^ inhibits hDAT [^3^H]DA uptake, Zn^2+^ instead increases hDAT T356M [^3^H]DA uptake (*p < 0.05 by one-way ANOVA followed by Dunnett’s test compared to 0 Zn^2+^ control; *n* = 4). **(B)** (*Top)* Representative AMPH-induced amperometric currents (reflecting DA efflux) are displayed in the presence or absence of 100 μM Zn^2+^. Arrows indicate the application of 10 μM AMPH. (*Bottom*) Maximal DA efflux amperometric current recorded in the presence of Zn^2+^ normalized to maximal current recorded in the presence of vehicle. (*p < 0.05 by paired Student’s *t*-test; *n* = 5). **(C)** (*Top*) Representative Zn^2+^-induced change in amperometric currents are displayed in response to 100 μM Zn^2+^ or vehicle control. Arrows indicate the application of 100 μM Zn^2+^. (*Bottom*) Change in amperometric current recorded in response to 100 μM Zn^2+^ or vehicle control (*p < 0.05 by paired Student’s *t*-test; *n* = 5).

CHO cells were transiently transfected with either wild-type hDAT or hDAT T356M. Cells were incubated with 50 nM [^3^H]DA at 37 °C for 5 min in the presence of varying concentrations of Zn^2+^. Consistent with previous reports [2,3], Zn^2+^ decreases the DA uptake rate for wild-type hDAT (Figure 1A, filled squares). In contrast, for hDAT T356M cells, Zn^2+^ increases DA uptake, partially reversing the functional deficit of this variant (Figure 1A, open circles).

### Zn^2+^ enhances AMPH-induced DA efflux in hDAT T356M

To specifically measure reverse transport, DA was loaded into the cytoplasm of hDAT T356M cells by a whole-cell patch clamp pipette held in current-clamp mode. This configuration supplies intracellular DA directly to the cell independent of forward transport by hDAT and allows the cell to control its membrane voltage. The whole-cell patch pipette was filled with an internal solution containing 2 mM DA as described previously [5]. DA efflux in response to 10 μM AMPH was measured by amperometry in the presence of 100 μM Zn^2+^ or vehicle control [5]. Representative amperometric traces are shown in Figure 1B (top). The peak of DA efflux normalized to vehicle control is shown in Figure 1B (bottom). Zn^2+^ increases AMPH-induced DA efflux in hDAT T356M compared to vehicle control (Figure 1B), indicating an enhancement of reverse transport DA in the presence of Zn^2+^. For comparison, Zn^2+^ partially rescues AMPH-induced DA efflux for hDAT T356M to a level of 48% ± 12% of wild-type hDAT AMPH-induced DA efflux [5].

### Zn^2+^ decreases baseline anomalous DA efflux in hDAT T356M

Using the whole-cell patch clamp technique coupled to amperometry, the effect of 100 μM Zn^2+^ on the baseline (anomalous) DA efflux of hDAT T356M was studied. The whole-cell patch pipette delivered intracellular DA into the cell in current-clamp mode. To determine the effect of Zn^2+^ on baseline DA efflux, the change in amperometric current was compared following the application of 100 μM Zn^2+^ or vehicle control. Baseline DA efflux decreased significantly in the presence of Zn^2+^ in comparison with that of vehicle, indicating that Zn^2+^ inhibits the constitutive DA efflux by hDAT T356M. Representative amperometric traces are shown in Figure 1C (top), and mean values are plotted in Figure 1C (bottom).

## Discussion

Here, we explore the potential for Zn^2+^ in rescuing the biophysical abnormalities in the hDAT variant T356M, which we recently reported in Hamilton et al. [5]. These functional deficits in hDAT T356M may contribute to the dysfunction in DA neurotransmission associated with ASD. Zn^2+^ was previously shown to partially restore DA uptake function in DAT mutant Y335A, which exhibits low uptake under basal conditions [7]. Here, we demonstrate that Zn^2+^ reverses deficits in both forward and reverse transport in the T356M variant. Additionally, Zn^2+^ decreases baseline anomalous DA efflux of the hDAT T356M *de novo* mutation, possibly providing an explanation for the positive effects of Zn^2+^ on the uptake and efflux properties of this mutant transporter. This is a novel and intriguing finding in terms of ameliorating irregularities in DAT function in a *de novo* ASD-associated mutation.

T356 is located in transmembrane domain 7, and the hDAT Zn^2+^ binding site spans the spatial micro-environment between the transmembrane helices 7 and 8 and extracellular loop 2 (EL2) [8,9]. Binding of Zn^2+^ to DAT alters the conformational equilibrium between the inward- and outward-facing state of the DAT [8,9]. However, mutation of an intracellular tyrosine to alanine (Y335A) converts the inhibitory Zn^2+^ switch into an activating Zn^2+^ switch, whereby Zn^2+^ rescues functions of the Y335A mutant transporter [7,8]. Therefore, the functional impact of Zn^2+^ binding to mutant transporters can be different than for wild-type hDAT as we observe here for T356M (Figure 1A). Whereas Zn^2+^ has been suggested to promote the outward facing conformation of wild-type hDAT, the structural effect of Zn^2+^ binding to hDAT T356M is unknown and remains an interesting topic for future investigation.

Clinical data have previously established that mean serum Zn^2+^ levels are significantly lower in children diagnosed with ASD compared to unaffected children and that there exist disturbances in Zn^2+^ metabolism in patients diagnosed with ASD [10-12]. hDAT T356M is the first *de novo* DAT mutation found in a patient with ASD, and hDAT T356M functional deficits can partially be rescued by Zn^2+^. Whether or not Zn^2+^ regulation of hDAT may be directly relevant for the etiology of ASD is presently unknown. However, our work suggests that the molecular mechanism engaged by Zn^2+^ to partially restore function in hDAT T356M may be a novel therapeutic target to rescue, at least in part, functional deficits in hDAT variants associated with ASD.

## Abbreviations

DAT: Dopamine transporter
hDAT: Human dopamine transporter
AMPH: Amphetamine
DA: Dopamine
ASD: Autism spectrum disorder
CHO: Chinese hamster ovary
